# Understanding the contribution of soybean crop residues inoculated with *Bradyrhizobium* spp. and not harvested on nitrogen supply in off-season corn cultivars

**DOI:** 10.1371/journal.pone.0269799

**Published:** 2022-06-22

**Authors:** Alan Mario Zuffo, Rafael Felippe Ratke, Mohammad K. Okla, Abdulrahman Al-Hashimi, Jorge González Aguilera, Amanda Camila Silva Trento, Natielly Pereira da Silva, Edicarlos Damacena de Souza, Bruna Karolayne Andrade Nogueira, Jéssica Heloiza Coutinho, Fábio Steiner, Francisco de Alcântara Neto, Gabriel Barbosa da Silva Júnior, Francisco Charles dos Santos Silva, Renato Lustosa Sobrinho, Hamada AbdElgawad

**Affiliations:** 1 Universidade Estadual do Maranhão, Campus de Balsas, Praça Gonçalves Dias, Balsas, MA, Brasil; 2 Universidade Federal do Mato Grosso do Sul, Chapadão do Sul, MS, Brasil; 3 Botany and Microbiology Department, College of Science, King Saud University, Riyadh, Saudi Arabia; 4 Universidade Federal de Rondonópolis, Rondonópolis, MT, Brasil; 5 Universidade Federal do Paraná, Curitiba, PR, Brasil; 6 Universidade Estadual do Mato Grosso do Sul, Unidade Universitária de Cassilândia, Cassilândia, MS, Brasil; 7 Departamento de Fitotecnia Ininga, Universidade Federal do Piauí, Campus Universitário Ministro Petrônio Portella, Teresina, PI, Brasil; 8 Department of Agronomy, Federal University of Technology, Pato Branco, PR, Brazil; 9 Department of Biology, Laboratory for Molecular Plant Physiology and Biotechnology, University of Antwerp, Antwerpen, Belgium; Federal University of Mato Grosso do Sul, BRAZIL

## Abstract

Excessive rainfall in the soybean preharvest period can make mechanized crop harvesting technically and economically unfeasible, causing 100% losses in soybean grain yield. An alternative to reduce the economic losses of farmers would be using unharvested soybean crop residues as a source of nitrogen (N) for the subsequent corn crop. However, a question that still needs to be understood is whether the amount of N released from unharvested soybean residues (straw and grains) is sufficient to meet all the nutritional demand for this nutrient in the off-season corn. Therefore, this study investigated the impact of unharvested soybean crop residue persistence on the yield response of off-season corn crop (*Zea mays* L.) to the application of N fertilizer rates when grown in tropical Cerrado soils of medium and high fertility. Four simple corn hybrids (SYN7G17 TL, 30F53VYHR, B2433PWU, and AG 8700 PRO3) were grown in soils of medium fertility and medium acidity level (UFMS 1) and high fertility and low acidity level (UFMS 2) and fertilized with five of N fertilizer rates (0, 40, 80, 120, and 160 kg ha^–1^ of N) applied at 30 days after emergence (DAE). Canonical correlation analysis (CCA) was used to investigate the interrelationships between the groups of independent (agricultural production areas, corn cultivars, and N application rates) and dependent (corn agronomic traits) variables. Crop residues remaining on the soil surface from soybeans not harvested and inoculated with Bradyrhizobium spp. can supply most of the nitrogen requirement of off-season corn grown in succession, especially in tropical soils of medium fertility. However, in high-fertility tropical soils, the maximum grain yield potential of off-season corn cultivars can be obtained with the application of mineral N fertilizer in supplement the amount of nitrogen released from unharvested soybean residues. Therefore, the N requirement depends on the corn cultivar and the agricultural production area. However, our results show that when off-season corn is grown on unharvested soybean residues, nitrogen fertilization in topdressing can be dispensed. The agricultural area with high fertility soil (UFMS 2) enhances the grain yield of the off-season corn crop. The corn cultivar AG 8700 PRO3 has a higher thousand-grain mass and high grain yield potential under Brazilian Cerrado conditions.

## Introduction

The Cerrado region occupies 203.4 million hectares of Central Brazil, representing approximately 24% of the Brazilian territory and 50% of the country’s agricultural area. The Cerrado currently produces approximately 98% of Brazil’s cotton (*Gossypium hirsutum* L.) production, 58% of its corn (*Zea mays* L.), and 55% of its soybeans [*Glycine max* (L.) Merrill.] [1]. Agricultural production in this region will certainly continue to be an important driver of Brazilian agribusiness in the coming decades. However, climate change has been identified as one of the greatest challenges for Brazilian and global agricultural production in this 21st century [2–5]. Among climate changes, the increase in the average temperature of the planet’s surface, changes in rainfall patterns, and the increase in the frequency of extreme weather events have had serious impacts on the world’s economy and agricultural sector [2, 3].

Agricultural production is highly dependent on environmental conditions, especially temperature and rainfall, and has often been limited by changes in weather patterns [3–5]. Recent studies have reported that the impact of excessive rainfall on crop yields can be similar to the impact of drought and extreme heat. Indeed, Yan et al. [5] showed that excessive rainfall reduced United States corn yields by up to 34%, while drought and excessive heat caused yield losses of up to 37%. Therefore, to maintain crop yields and ensure food production stability, farmers will need a suite of adaptation strategies, and the choice of strategy will depend on how the local to regional climate is expected to change [4].

Recent flooding caused by excessive rainfall during the rainy season in the Brazilian Cerrado region has brought researchers’ attention to the complex agricultural problems associated with global climate change [6]. Continued rainfall and excessively wet field conditions, particularly in January and February coinciding with the end of the soybean cycle (maturation) in the Cerrado region, have often delayed the start of mechanized soybean harvesting in recent years [7, 8]. However, heavy rains in some Cerrado regions have left many soybean fields in flooded or saturated soil conditions, which has impeded mechanized harvesting and caused 100% losses in soybean yield. Excessive rainfall in the soybean preharvest period leads to significant crop yield losses by causing direct damage to the qualitative, quantitative, and sanitary aspects of soybean seeds [5, 8, 9]. In extreme situations, the exposure of soybean plants to prolonged periods of rainfall and high moisture content of grains makes mechanized harvest technically and economically unfeasible due to the low physical and sanitary quality of the seeds caused by deterioration and fermentation of soybean grains [8, 10].

An alternative to reduce the economic losses of farmers due to the impediment of the soybean harvest caused by extreme weather events during the harvest period would be the use of soybean plant residues as a source of nitrogen (N) for the subsequent second corn crop ("safrinha" or "out of season corn"). Soybeans require large amounts of N throughout their development cycle. Approximately 80 kg of N is needed for each ton of soybean grain produced [11], which is fulfilled by biological N fixation (BNF) through symbiosis with strains of *Bradyrhizobium* spp. and by N uptake from the soil or applied with N fertilizers [12, 13]. Therefore, residues from soybean plants (straw and grains) have a high N content when compared to the straw of grass plants, and these residues breakdown more easily, releasing N into the soil. However, the potential of soybean when this summer leguminous crop is not harvested to reduce the N requirement of the off-season corn has not yet been documented.

The use of simple corn hybrids in off-season corn cultivation between January and May (after the summer soybean crop) has high yield potential in the Cerrado region. However, to express all this production potential, the application of high rates of N fertilizers is required [14, 15]. In many situations, reduced rates of N fertilizers have been applied when off-season corn is grown after the soybean crop [16]. The lower N rates have been justified by the low response of off-season corn to N fertilization due to the higher N input in the soil provided by the soybean crop.

To adjust N fertilization for this crop succession in the Brazilian Cerrado region, it has been considered that each ton of soybean grain corresponds to 17 kg ha^–1^ of N in the crop residue, which is released for the subsequent corn crop [15]. Therefore, an average yield of 3.6 Mg ha^–1^ of soybeans would indicate a surplus of up to 61 kg ha^–1^ of N, which is insufficient to meet the N demand of off-season corn [14]. However, an issue that still needs to be understood is the proper management of N fertilization when corn is grown after unharvested soybeans. A question that we need to answer is "the amount of N released from the residues of unharvested soybean plants (straw and grains) sufficient to meet all the nutritional demand for this nutrient in the off-season corn crop". This issue is very important in the current scenario of world agricultural production when we consider the high prices of mineral fertilizers in this last agricultural season [17]. Therefore, the application of reduced rates of N or even the nonapplication of N fertilizers is an alternative to reduce production costs and improve the farmer’s profitability with the off-season corn crop.

Corn crops grown after unharvested soybeans can benefit from the fast and greater release of nutrients from decomposing legume crop residues, which have a low C/N ratio [15]. However, the N demand of the off-season corn crop depends on the cultivar, crop succession system, and the interaction of the genotype with the production environment [14]. For instance, the economically optimum N rate reported for off-season corn grown in the Cerrado region ranged from 40 to 180 kg ha^−1^ of N, depending on the production system and the year [14, 15, 18–20]. Therefore, N has been considered the nutrient with higher yield response for off-season corn crops grown in tropical soils. However, the response of off-season corn grown on the residues of the unharvested soybean crop has not yet been documented.

This study investigated the impact of unharvested soybean crop residue persistence on the yield response of four off-season corn hybrids (*Zea mays* L.) to application of N fertilizer rates when grown in tropical Cerrado soils of medium and high fertility.

## Material and methods

### Study site description

**Fig 1:**
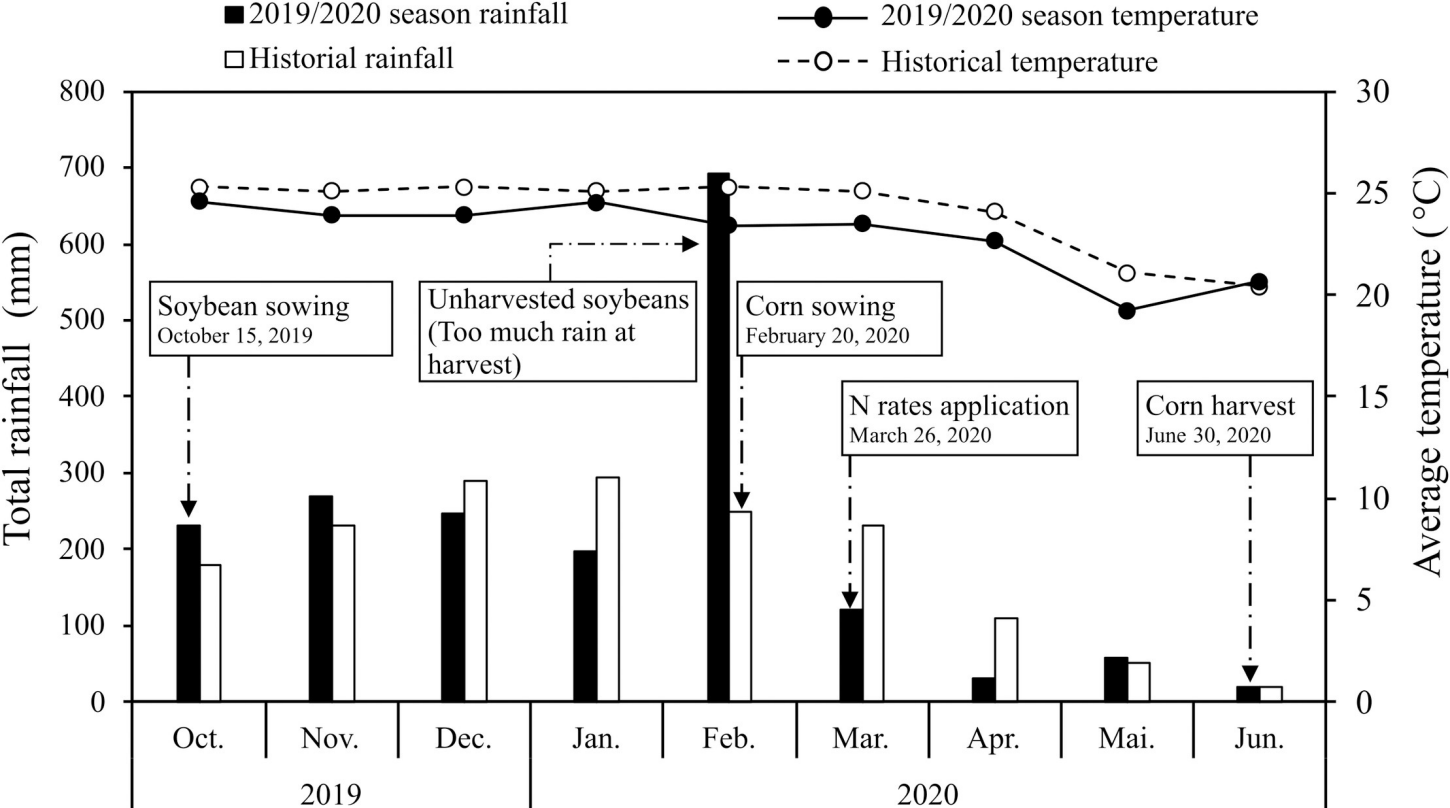
Monthly total rainfall (bars) and monthly average temperature (lines) in Chapadão do Sul, Mato Grosso do Sul, Brazil, during soybean and corn cropping in the 2019/2020 season and 30-yr historical average data (1990 to 2020). Data were accessed through the National Meteorological Institute database (https://bdmep.inmet.gov.br/).

The field experiments were conducted in two areas of the agricultural experiment station of the Federal University of Mato Grosso do Sul, called UFMS 1 and UFMS 2, in Chapadão do Sul, MS, Brazil (18°46′18″S, 52°37′25″W with an altitude of 810 m), during the 2019/2020 growing season. The regional climate, according to the Köppen classification, is Aw, characterized as tropical with hot summers and high rainfall rates and dry winters, with a dry season between May and September. The mean annual temperature is 24.0°C, with a minimum of 18.8°C (July) and a maximum of 29.0°C (January). The mean annual rainfall is 1,850 mm. The climatic conditions recorded during the experiments are shown in Fig 1. The total rainfall in February 2020 was nearly three times the 30-year historical average.

The soils of the agricultural experimental stations were classified as Rhodic Hapludox [21], or "Latossolo Vermelho" in the Brazilian soil classification [22]. The occurrence of Rhodic Hapludox in the northeast region of Mato Grosso do Sul state is common, and this class of soil has no restrictions with respect to agricultural use and management [22]. The two agricultural experimental areas were cultivated under no-till conditions for two years with soybean/corn rotations in the 2017/2018 and 2018/2019 growing seasons. Before starting the experiments, soil samples were collected from the 0–0.20 m layer using a hole auger at five points per plot. The results of the soil chemical analysis did not show significant variability between the plots with respect to the soil chemical properties of the experimental area. The chemical analysis showed that the soils have medium fertility and medium acidity levels (UFMS 1) and high fertility and low acidity levels (UFMS 2), which allowed adequate availability of nutrients to plants. The average values of the soil chemical analysis are shown in Table 1.

**Table 1:** Soil chemical properties of the two agricultural experimental areas.

Agricultural area	pH	OM	P_Mehlich-1_	H+Al	Al^3+^	Ca^2+^	Mg^2+^	K^+^	CEC	V
	CaCl_2_	g dm^–3^	mg dm^–3^	------------------- cmol_c_ dm^–3^ ---------------------						%
UFMS 1	5.3	27.3	14.1	3.70	0.20	2.70	0.80	0.30	7.50	51
UFMS 2	5.7	35.3	41.6	3.90	0.05	4.20	1.60	0.45	10.15	62

OM: Organic matter. CEC: Cation exchange capacity at pH 7.0. V: Soil base saturation.

### Experimental design and treatments

The treatments were arranged as split-split plots in an randomized complete block design with three replicates. In the main plots, two agricultural production areas with distinct levels of fertility were established. The first production area had medium-fertility soil and was named UFMS 1, while the second production area had high-fertility soil and was named UFMS 2 (see Table 1). The subplots were represented by the cultivation of four corn cultivars SYN7G17 TL (early cycle single hybrid), 30F53VYHR (early cycle single hybrid), B2433PWU (super early cycle single hybrid), and AG 8700 PRO3 (early cycle simple hybrid). In the subsubplots, five N fertilization rates (0, 40, 80, 120, and 160 kg ha^–1^ of N) were applied at 30 days after emergence (DAE). Nitrogen fertilizer topdressing was carried out at the V4 growth stage when the corn plants had four fully expanded leaves with visible collar. Urea (45% N) was used as the N source. The experimental units consisted of seven 5.0-m long rows, with 0.45 m between rows. The useful area comprised the three central rows of each subsubplot, disregarding 1.0 m of each edge (i.e., 4.05 m^2^).

### Plant material, fertilization, and crop management

In October 2019, the soybean crop was sown in both agricultural production areas. Soybean was sown mechanically in rows 0.45 m apart at a density of 13 seeds per meter (i.e., 260,000 to 280,000 plants per hectare). Base fertilization was carried out by applying 78 kg ha^–1^ of P_2_O_5_ and 16 kg ha^–1^ of N (monoammonium phosphate—MAP) at the sowing furrow. At 30 days after sowing [V4 stage—four fully expanded leaves (fourth trifoliolate)], 80 kg ha^–1^ of K_2_O (KCl) was applied in topdressing. Soybean fertilization was carried out according to the crop requirements for cultivation in Cerrado tropical soils [23].

All soybean seeds used in the experiment were previously inoculated with efficient *Bradyrhizobium* spp. strains. The commercial liquid inoculant Simbiose Nod Soja^®^ (Simbiose: Biological Agrotechnology), containing the *Bradyrhizobium japonicum* strains [CPAC-15 (SEMIA 5079)] and *Bradyrhizobium diazoefficiens* strains [CPAC-7 (SEMIA 5080)] (minimum concentration of 7.2 × 10^9^ viable cells per mL) was used at a rate of 3.0 mL kg^–1^ seed as recommended by the manufacturer.

At the R7.2 growth stage (when 70% of the pods reached their mature pod color), the soybean plants were desiccated with 2 L ha^–1^ Gramoxone 200^®^ [200 g L^–1^ of 1,1’-dimethyl-4,4’-bipyridinium (Paraquat)] at a spray volume of 160 L ha^–1^. However, soybeans were not harvested due to the high incidence of rainfall that prevented mechanized harvesting of the crop (a situation that commonly prevents mechanized harvesting of soybeans in the Brazilian Cerrado region in years with high rainfall during the summer, especially in January and February). The average preharvest soybean yield was estimated at approximately 3,200 kg ha^–1^. At 15 days after the desiccation of the soybean plants, the corn crop was sown on the soybean crop residues (straw and grain) in a no-tillage system (NTS).

The corn crop was mechanically sown on February 20^th^, 2020, at a depth of 5.0 cm, in rows 0.45 cm apart at a density of four seeds per meter to reach a final stand of 70,000 to 75,000 plants per hectare. Base fertilization consisted of 104 kg ha^–1^ of P_2_O_5_ and 22 kg ha^–1^ of N [monoammonium phosphate (MAP)] applied at the sowing furrow. Corn seeds were previously treated with 70 g ha^–1^ cyantraniliprole, 0.6 g ha^–1^ metalaxyl-M, 4.5 g ha^–1^ thiabendazole and 0.75 g ha^–1^ fludioxonil.

At 30 DAE of corn plants (V_4_ stage–four fully expanded leaves with visible collar), 80 kg ha^–1^ of K_2_O (KCl) and treatments with different N fertilizer rates (ureia) were applied in topdressing. At 40 DAE (V_6_ stage–six fully expanded leaves with visible collar), foliar fertilization was applied using 1 L ha^–1^ of Actilase ZM^®^ (50 g L^–1^ of Zn, 42 g L^–1^ of S, and 30 g L^–1^ of Mn). Corn fertilization was carried out according to the crop requirements for cultivation in Cerrado tropical soils [23].

Weed management was performed at 20 DAE, using 1.5 L ha^–1^ of atrazine and 100 mL ha^–1^ of tembotrione. At the beginning of corn flowering, crop disease management was carried out with the application of 100 g ha^–1^ pyraclostrobin and 37.5 g ha^–1^ epoxiconazole. Pest management was carried out with the application of 130 g ha^–1^ methomyl, 30 g ha^–1^ of acetamiprid, and 30 g ha^–1^ bifenthrin. The application of these products was carried out according to the recommendations for the registered commercial products.

### Sampling of soybean crop residues

At 15 days after desiccation of the soybean crop, the amount of above-ground plant residues (straw and grain) was determined by taking samples from three random points per subsubplot using a 0.25 m^2^ (0.50 × 0.50 m) metal frame. The collected plant material was then oven-dried at 60°C (± 2°C) for five days, weighed, ground, and the N concentration was determined by the Kjeldahl method with digestion in sulfuric acid solution and vapor distillation as described by Silva [24]. Considering the nutrient concentration and dry matter produced, the total amount (kg ha^–1^) of N accumulated in soybean crop residues was calculated.

### Measurement of plant nutrition and corn production

At the flowering stage, after the emergence of corn female inflorescence, a total of 10 leaves opposite and below the ear were collected per experimental unit for the determination of N concentration. The leaf samples were dried in an oven at 60°C for 72 h and then ground in a Willey mill equipped with a 40-mesh sieve. After digestion with sulfuric acid, the leaf N concentration was determined by the semimicro Kjeldahl method [24].

At physiological maturity (R6 growth stage), plant height and ear insertion height were measured in ten plants per subsubplot. The plant height (cm) was measured from the soil surface to the insertion of the last leaf using a tape measure. The ear insertion height (cm) was determined from the soil surface to the insertion of the first ear using a tape measure.

The ears of corn in the subsubplots useful area were manually harvested on June 30^th^, 2020, and then threshed in a Wintersteiger Classic Plot Harvester. The grain yield and agronomic traits were then measured. The ear length, the number of grain rows per ear, the number of grains per row, and ear diameter were determined in twenty ears chosen at random. The mass of one thousand grains (g) was determined by the average of five measurements of 100 grains taken at random. The grain yield (kg ha^–1^) was estimated after the correction of grain weights to 13% moisture. The grain protein concentration was determined by the semimicro Kjeldahl method as recommended by Silva [24].

### Statistical analysis

The data were previously submitted to the statistical hypothesis verification tests of homoscedasticity of variances (Levene test; p > 0.05) and normality of residues (Shapiro–Wilk test; p > 0.05). Then, the data were submitted to analysis of variance (ANOVA) following the statistical model of the split-split plot (SSP) and randomized complete block design (RCBD). The means of the qualitative factors (agricultural production areas or corn cultivars) were compared by the LSD test at the 0.05 level of confidence. Regression analysis was used for the N fertilizer application rates (quantitative factor), and significance equations (F test, p ≤ 0.05) were adjusted according to the highest coefficients of determination. These analyses were performed using Sisvar^®^ software, version 5.6 for Windows (Statistical Analysis Software, UFLA, Lavras, MG, BRA).

Canonical correlation analysis (CCA) was used to study the interrelationship between sets (vectors) of independent (agricultural production area, corn cultivars, and N application rates) and dependent (corn morphoagronomic traits) variables. These analyses were performed using RBio software version 140 for Windows (Rbio Software, UFV, Viçosa, MG, BRA).

## Results and discussion

### Dry matter and nitrogen accumulated in soybean crop residues

The total amount of soybean crop residues remaining on the soil surface at the time off-season corn was sown was 5,314±576 kg ha^–1^ and 5,915±531 kg ha^–1^ in agricultural areas of medium fertility (UFMS 1) and high fertility (UFMS 2), respectively (Table 2). In the agricultural area with medium fertility soil, soybean straw (dead leaves, stems, and pods) and grains represented 45.9% (2,439 kg ha^–1^) and 54.1% (2,876 kg ha^–1^) of the total crop residues remaining on the soil surface, respectively. In the agricultural area of high fertility (UFMS 2), soybean straw and grains represented 46.9% (2,772 kg ha^–1^) and 53.1% (3,143 kg ha^–1^) of the total crop residues remaining on the soil surface, respectively (Table 2). This amount of soybean crop residues remaining on the soil surface is important for the maintenance and improvement of the no-tillage system. The input of adequate amounts of crop residues on the soil surface favors soil organic matter accumulation, nutrient cycling and increases crop yields [25, 26].

**Table 2:** Dry matter production, N concentration and accumulation of unharvested soybean crop residues grown in tropical Cerrado soil of medium (UFMS 1) and high fertility (UFMS 2). Chapadão do Sul, MS, Brazil.

Agricultural area	Dry matter (kg ha^–1^)			N concentration (g kg^–1^)		N accumulation (kg ha^–1^)		
	Straw	Grain	Total	Straw	Grain	Straw	Grain	Total
**UFMS 1**	2,439±280	2,876±296	5,314±576	25.1±1.4	55.7±5.5	61±7.4	160±17.8	221±25.2
**UFMS 2**	2,772±142	3,143±389	5,915±531	24.7±2.8	57.2±2.7	68±8.1	180±9.7	248±17.8

The N concentrations in soybean straw and grains ranged from 24.7 to 25.1 g kg^–1^ and 55.7 to 57.2 g kg^–1^, respectively (Table 2). Nitrogen concentrations ranging from 25 to 30 g kg^–1^ is usually reported for soybean crop residues [27], while the N concentration observed in soybean grains commonly ranges from 55 to 70 g kg^–1^ [13]. Considering that 37% of the dry matter of soybean crop residues consists of carbon [27], the C:N ratio of soybean residues remaining on the soil surface is 15:1. Crop residues with high N concentrations and low C:N ratio (< 20:1), such as soybean crop residues, are readily decomposed by soil microorganisms due to the lower straw recalcitrance, resulting in a fast release of nutrients to the subsequent crop [15, 25]. Indeed Gezahegn et al. [27] showed that approximately 75% of total N was released during the first 45 days after soybean harvest.

The total amount of N accumulated in soybean crop residues remaining on the soil surface was 221±25.2 kg ha^–1^ and 248±17.8 kg ha^–1^ in agricultural areas of medium fertility (UFMS 1) and high fertility (UFMS 2), respectively (Table 2). In general, the amount of N accumulated in straw (dead leaves, stems, and pods) and soybean grains represented 27.5% (from 61 to 68 kg ha^–1^ N) and 72.5% (from 160 to 180 kg ha^–1^ N) of the total N accumulated crop residues remaining on the soil surface, respectively.

These results show the large increase in N input in the production system for the subsequent off-season corn crop when the soybean crop cannot be harvested due to excessive rainfall at harvest. The exposure of soybean crops to prolonged periods of rainfall during preharvest makes mechanized harvest technically and economically unfeasible due to the low physical and sanitary quality of the seeds caused by deterioration and fermentation of soybean grains [8, 10]. Therefore, in years subject to extreme weather conditions, unharvested soybean residues deposited on the soil surface can result in a surplus of up to 180 kg ha^–1^ of N for the subsequent crop. Underthese cropping conditions, unharvested soybean residues are an important source of N for off-season corn crops. The N application rate in this crop should be adjusted to improve the efficiency of N fertilizer use and limit soil N losses by leaching [14, 16, 18].

### Nitrogen nutrition, production components, and corn yield

The results of the analysis of variance showed significant effects (p < 0.05) for the interaction between the agricultural production area, corn cultivars, and N application rates for the leaf N concentration and grain protein concentration (Table 3). Significant effects (p < 0.05) were observed for the interaction between agricultural production area and corn cultivars for ear length and diameter (Table 3). The interaction between agricultural production area and N application rates was significant for thousand-grain mass and grain yield. There was an isolated and significant effect of the agricultural production area on the ear insertion height and number of grain rows per ear. The effect of corn cultivars was significant for leaf N concentration, ear diameter, number of grain rows per ear, number of grains per row, thousand-grain mass, grain yield, and grain protein concentration. Nitrogen application rates had a significant effect only on leaf nitrogen concentration.

**Table 3:** Probability values of the variance analysis test for the measurements of grain yield and morphoagronomic traits of corn cultivars for the effects of agricultural production areas, cultivars, and N application rates during the 2020 growing season in Chapadão do Sul, MS, Brazil.

Sources of variation	Probability > F								
	N	EIH	EL	ED	NRE	NGR	M1000	GY	GPC
**Agricultural area (A)**	0.186	**0.022**	0.230	0.605	**0.037**	0.741	0.171	0.580	0.322
**Corn cultivar (C)**	<0.01	0.118	0.169	<0.01	0.201	0.134	0.101	0.121	0.041
**Nitrogen rates (N)**	<0.01	0.480	0.660	0.997	0.066	0.728	0.317	0.822	0.060
**A × C**	0.033	0.415	**0.031**	**0.041**	0.481	0.262	0.484	0.227	<0.01
**A × N**	<0.01	0.420	0.258	0.213	0.429	0.227	**<0.01**	**<0.01**	<0.01
**C × N**	<0.01	0.608	0.143	0.597	0.080	0.755	0.486	0.297	<0.01
**A × C × N**	**<0.01**	0.326	0.594	0.881	0.906	0.862	0.203	0.525	**<0.01**
**CV**_**Plot**_ **(%)**	16.27	4.98	9.82	6.50	4.11	17.31	17.95	40.86	15.08
**CV**_**Subplot**_ **(%)**	9.64	17.42	15.20	5.17	5.57	12.46	8.91	27.38	10.35
**CV**_**Sub subplot**_ **(%)**	9.59	7.65	10,80	3.71	4.88	8.71	12.65	13.92	10.78

N: leaf nitrogen concentration; EIH: ear insertion height; EL: ear length; ED: ear diameter; NRE: number of grain rows per ear; NGR: number of grains per row; M1000: thousand-grain mass; GY: grain yield; GPC: grain protein concentration. CV: coefficient of variation.

The highest leaf N concentration was observed in cultivar 30F53VYHR in both agricultural production areas (UFMS 1 and UFMS 2) (Table 4). In the high fertility agricultural area (UFMS 2), the cultivar AG8700 PRO3 had the lowest leaf N concentration compared to the other corn cultivars. The leaf N concentrations of cultivars 30F53 VYHR and AG 8700 PRO3 were significantly higher in the medium fertility agricultural area (UFMS 1). Corn cultivars grown in contrasting production environments commonly have different uptake capacities and N use efficiencies [14, 16, 28–30]. These characteristics are related to the morphological characteristics of the roots of N-efficient and N-inefficient corn cultivars [30].

**Table 4:** Leaf nitrogen concentration and grain protein content of off-season corn cultivars grown under unharvested soybean crop residues in tropical Cerrado soils of medium (UFMS 1) and high fertility (UFMS 2) during the 2020 growing season. Chapadão do Sul, MS, Brazil.

Agricultural area	Corn cultivar			
	SYN7G17 TL	30F53 VYHR	B2433 PWU	AG8700 PRO3
	**Leaf nitrogen concentration (g kg** ^**-1**^ **)**			
**UFMS 1**	37.7±3.0 aB	44.5±0.5 aA	36.1±1.4 aB	36.1±0.5 aB
**UFMS 2**	36.8±0.4 aB	40.0±0.4 bA	37.0±0.4 aB	31.8±0.2 bC
	**Grain protein concentration (%)**			
**UFMS 1**	12.2±0.3 aA	11.2±0.7 aB	11.4 ±0.1 aB	11.1±3.3 aB
**UFMS 2**	10.5±0.4 bA	10.2±0.1 bA	9.4 ±0.3 bB	10.7±0.4 aA

Means followed by distinct lowercase letters for the agricultural production areas (in the column) or distinct uppercase letters for the corn cultivars (in the line) show significant differences (LSD test, p ≤ 0.05).

The cultivar SYN7G17 TL has a higher protein content in the grains than the other corn cultivars grown in the UFMS 1 agricultural area (Table 4). The lowest grain protein content was observed in the B2433 PWU cultivar grown in the UFMS 2 agricultural area. The cultivation of corn cultivars SYN7G17 TL, 30F53 VYHR, and B2433 PWUA in the medium fertility agricultural area (UFMS 1) resulted in the highest grain protein concentration when compared to the UFMS 2 agricultural area.

The significant effect of the interaction between nitrogen application, corn hybrids and agricultural production environments on grain protein content has been commonly reported in other studies [31]. The significance of corn hybrids indicates the existence of genetic variability and the interaction between genotypes, production environments and N application rates demonstrates the importance of studies with these three factors. Furthermore, the results of these studies help to better understand the efficiency, absorption, and translocation of N from corn plants to grains. The leaf N concentration observed in this study for most cultivars and agricultural areas exceeded the range of 28 to 35 g kg^–1^ (Table 4), which is considered adequate for corn crops [23]. These results reflect the high input of N in the soil from the unharvested soybean residues (Table 2) and N fertilizer rates applied. Nitrogen is a structural component of chlorophyll, amino acids, and protein molecules and is essential for the establishment and duration of the leaf area, as well as for the formation of grains and ears of corn. Therefore, N can interfere both in the magnitude of the source producing photoassimilates (photosynthesis) and in the strength of the drain that will receive them [32].

Nitrogen application rates resulted in distinct responses of corn cultivars to leaf N concentration and grain protein concentration in both areas of agricultural production (Table 5). In the medium fertility agricultural area (UFMS 1), the B2433 PWU and AG8700 PRO3 cultivars had a significant response to leaf N concentrations up to rates of 98.8 and 58.2 kg ha^–1^ of N, respectively. On the other hand, the applied N rates did not significantly affect the N concentration of the cultivars SYN7G17 TL and 30F53 VYHR (Table 5). In the high fertility agricultural area (UFMS 2), the applied N rates resulted in a linear increase in the leaf N concentration of the cultivar 30F53 VYHR, while the leaf N concentration of the cultivar SYN7G17 TL linearly decreased with N rates. On the other hand, the highest leaf N concentration of the cultivar AG8700 PRO3 was obtained with the application of 80.0 kg ha^–1^ of N, and the leaf N concentration of cultivar B243 3PWU was not significantly affected by the applied N rates.

**Table 5:** Response to nitrogen (N) application rates of four off-season corn cultivars grown under unharvested soybean crop residues in tropical Cerrado soils of medium (UFMS 1) and high fertility (UFMS 2) during the 2020 growing season on the leaf nitrogen concentration and grain protein concentration. Chapadão do Sul, MS, Brazil.

Agricultural area	Corn cultivar	Regression equations	R^2^	MER (kg ha^–1^)	MEV
		**Leaf nitrogen concentration (g kg** ^**–1**^ **)**			
UFMS 1	SYN7G17 TL	ŷ = ӯ = 37.7	0.08^NS^	–	37.7
	30F53 VYHR	ŷ = ӯ = 44.5	0.01^**NS**^	–	44.5
	B2433 PWU	ӯ = 28.3 + 0.257x – 0.0013x^2^	0.80*	98.8	41.0
	AG8700 PRO3	ӯ = 36.1 + 0.163x – 0.0014x^2^	0.82*	58.2	40.8
UFMS 2	SYN7G17 TL	ŷ = 46.0–0.115x	0.44*	0	46.0
	30F53 VYHR	ŷ = 35.7 + 0.066x	0.49*	160.0	45.3
	B243 3PWU	ŷ = ӯ = 36.9	0.20^**NS**^	–	36.9
	AG8700 PRO3	ŷ = 28.9 + 0.128x – 0.0008x^2^	0.61*	80.0	34.0
		**Grain protein concentration (%)**			
UFMS 1	SYN7G17 TL	ŷ = 10.76 + 0.021x	0.55*	160.0	14.1
	30F53 VYHR	ŷ = ӯ = 12.25	0.08^**NS**^	–	12.2
	B2433 PWU	ŷ = 9.90 + 0.021x	0.48*	160.0	13.3
	AG8700 PRO3	ŷ = ӯ = 11.22	0.01^**NS**^	–	11.2
UFMS 2	SYN7G17 TL	ŷ = 8.31 + 0.027x	0.62*	160.0	12.6
	30F53 VYHR	ŷ = 11.61–0.016x	0.58*	0	11.6
	B243 3PWU	ŷ = ӯ = 9.42	0.11^**NS**^	–	9.4
	AG8700 PRO3	ŷ = 13.46–0.034x	0.76*	0	13.5

*: Significant (F-test, p ≤ 0.05). ^NS^: Non-significant (F-test, p > 0.05). R^2^: determination coefficient. MER = maximum estimated rate. MEV = maximum estimated value.

Nitrogen application rates in the UFMS 1 agricultural area resulted in a linear increase in the grain protein concentration of the SYN7G17 TL and B2433 PWU cultivars, while the grain protein concentration of the 30F53 VYHR and AG8700 PRO3 cultivars was not significantly affected by N application rates. In the high fertility agricultural area (UFMS 2), the grain protein concentration increased linearly with the N rates applied in the corn cultivar SYN7G17 TL, while the grain protein concentration of the 30F53 VYHR and AG8700 PRO3 cultivars linearly decreased with N rates (Table 5).

In both areas of agricultural production, the corn cultivar AG8700 PRO3 had a significant increase in leaf N concentration with N fertilizer rates. However, this higher N concentration in the corn plants with N fertilization did not result in a significant effect on the grain protein content in the medium fertility area (UFMS 1) and resulted in a decrease in the grain protein content in the high fertility agricultural area (UFMS 2). It was expected that the increase in nitrogen uptake by cultivar AG8700 PRO3 with the application of N would also result in greater protein accumulation in corn grains. However, we emphasize that the corn crop was cultivated under residues of the unharvested soybean crop, which resulted in a large input of this nutrient to the soil (from 221 to 248 kg ha^–1^ of N, see Table 2). Therefore, most of the N requirement of corn plants was supplied by N released from unharvested soybean residues. This high input of N in the soil resulted in the lack of response of some corn cultivars to N fertilization in both agricultural production areas (Table 5). These results indicate that the increase in leaf N concentration and grain protein content in off-season corn grown under unharvested soybean residues is more dependent on the N demand of the genotype than on the agricultural production system.

The soil fertility level of the two agricultural production areas did not significantly affect (p < 0.05) the length and diameter of ears in all corn cultivars, except for the ear length of cultivar AG8700 PRO3 (Table 6). The medium fertility agricultural area resulted in the ear of the cultivar AG 8700 PRO3 having a greater length than the agricultural area of high fertility. The B2433PWU and AG 8700 PRO3 cultivars have a larger ear diameter than the other corn cultivars in both agricultural production areas. Such findings are related to the genetic characteristics of corn hybrids and their interaction with the production system. The length and diameter of ears of corn are characteristics that directly affect the number of grains per ear and the grain yield of the crop [14, 20].

**Table 6:** Ear length and ear diameter of off-season corn cultivars grown under unharvested soybean crop residues in tropical Cerrado soils of medium (UFMS 1) and high fertility (UFMS 2) during the 2020 growing season. Chapadão do Sul, MS, Brazil.

Agricultural area	Corn cultivar			
	SYN7G17 TL	30F53 VYHR	B2433 PWU	AG8700 PRO3
	**Ear length (cm)**			
**UFMS 1**	14.81±0.87 aA	13.88±0.34 aA	14.91± 0.28 aA	17.07± 0.50 aA
**UFMS 2**	14.22± 0.60 aA	15.00± 0.24 aA	15.31± 0.30 aA	14.32±0.29 bA
	**Ear diameter (mm)**			
**UFMS 1**	44.88±0.59 aB	42.83±0.53 aB	49.70±0.48 aA	50.51±0.50 aA
**UFMS 2**	44.40±0.33 aB	44.44±0.36 aB	49.76±0.52 aA	47.97±0.53 aA

Means followed by distinct lowercase letters, for the agricultural production areas (in the column) or distinct uppercase letters, for the corn cultivars (in the line) show significant differences (LSD test, p ≤ 0.05).

The thousand grain mass decreased linearly with the N rates applied in the medium fertility agricultural area (UFMS 1), while the N fertilization did not significantly affect the thousand grain mass of corn cultivated in the high fertility agricultural area (UFMS 2) (Table 7). The highest grain yield in the medium fertility area (UFMS 1) was obtained with the application of 47.5 kg ha^–1^ of N, while the N application rates resulted in a linear increase in corn grain yield in the high fertility area (UFMS 2). These results show that even if the thousand grain mass is reduced by N fertilization, the corn crop has a positive response in grain yield up to the rate of 47.5 kg ha^–1^ of N in the agricultural area with medium fertility soil. On the other hand, a higher grain yield in the high-fertility area can be obtained with the application of high rates of N fertilizer (Table 7). The corn response to higher N fertilizer rates in the high fertility agricultural area, when compared to the medium fertility area, may be due to the higher level of soil fertility (Table 1). The greater availability of other nutrients in the UFMS 2 area, such as P, Ca, Mg, and K, must have improved the uptake of N from the soil, resulting in a higher grain yield response in the off-season corn crop. The crop yield response to N fertilization can be affected indirectly by the greater supply of other nutrients to plants, especially P and K [32, 33]. Therefore, the greater availability of nutrients in the UFMS 2 agricultural area resulted in greater plant development and a greater crop yield response (Table 7).

**Table 7:** Effects of nitrogen (N) application rates on thousand-grain mass and grain yield of off-season corn crop grown under unharvested soybean crop residues in tropical Cerrado soils of medium (UFMS 1) and high fertility (UFMS 2) during the 2020 growing season. Chapadão do Sul, MS, Brazil.

Agricultural area	Regression equations	R^2^	MER (kg ha^–1^)	MEV
	**Thousand-grain mass (g)**			
UFMS 1	ŷ = 156.0–0.108x	0.96*	0	156
UFMS 2	ŷ = ӯ = 157.4	0.31^NS^	–	157
	**Grain yield (kg ha** ^**–1**^ **)**			
UFMS 1	ŷ = 4,313 + 3.80x – 0.04x^2^	0.76*	47.5	4,403
UFMS 2	ŷ = 4,339 + 3.86x	0.95*	160.0	4,957

*: Significant (F-test, p ≤ 0.05). ^NS^: Non-significant (F-test, p > 0.05). R^2^: determination coefficient. MER = maximum estimated rate. MEV = maximum estimated value.

The yield responses of off-season corn to nitrogen fertilization in the Cerrado region have been very variable with the production environment and the genotype used. Simão et al. [18] showed that the application of 50 kg ha^–1^ of N at the V3 growth stage, in addition to the fertilization of 30 to 50 kg ha of N at the sowing base resulted in the highest grain yield of off-season corn in the Cerrado region. In general, the economically optimum N rate reported for off-season corn grown in the Cerrado region ranged from 40 to 180 kg ha^−1^ of N, depending on the production system and the year [14, 15, 20].

The ear insertion height was significantly (p < 0.05) higher in the medium fertility agricultural area (UFMS 1), while the highest number of grain rows per ear was obtained in the high fertility area (UFMS 2) (Table 8). A higher ear insertion height is desirable to facilitate the mechanized harvesting of corn crops [20]. In turn, the greater number of grain rows per ear results in the highest number of grains per ear and, consequently, in the higher grain yield, as observed for corn grown in the high fertility agricultural area (Table 7).

**Table 8:** Ear insertion height (EIH) and number of grain rows per ear (NRE) of off-season corn crops grown under unharvested soybean crop residues in tropical Cerrado soils of medium (UFMS 1) and high fertility (UFMS 2) during the 2020 growing season. Chapadão do Sul, MS, Brazil.

Agricultural production area	EIH (cm)	NRE (units)
**UFMS 1**	99.11±1.13 a	15.96±0.15 b
**UFMS 2**	93.35±1.48 b	16.57±0.16 a

Means followed by distinct letters show significant differences (F test, p ≤ 0.05).

The number of grain rows per ear was significantly (p < 0.05) higher in the B2433 PWU and AG8700 PRO3 cultivars (Table 9). The highest number of grains per row was observed in the cultivar 30F53VYHR. In turn, the corn cultivar AG 8700 PRO3 had the highest thousand-grain mass and grain yield when compared to the other cultivars. All cultivars used in this study are simple hybrids, which have high yield potential. However, the expression of this high yield potential depends on the interaction of the genotype with the growing environment. However, the grain yield of the cultivar AG 8700 PRO3 was 40% higher than the grain yield of the SYN7G17 TL and 30F53 VYHR cultivars (Table 9). This corn cultivar has some important agronomic characteristics and high yield potential, making it an excellent alternative for off-season corn cultivation for Brazilian farmers.

**Table 9:** Number of grain rows per ear (NRE), number of grains per row (NGR), one thousand grain mass (M1000), and grain yield (GY) of four off-season corn cultivars grown under unharvested soybean crop residues in tropical Cerrado soils during the 2020 growing season. Chapadão do Sul, MS, Brazil.

Corn cultivars	NRE (units)	NGR (units)	M1000 (g)	GY (kg ha^–1^)
**SYN7G17 TL**	15.82±0.17 b	26.80±0.50 b	139.15±3.39 b	4039±170.35 b
**30F53 VYHR**	15.68±0.10 b	29.12±0.55 a	141.03±4.45 b	3984±140.94 b
**B2433 PWU**	17.72±0.19 a	26.38±0.39 b	134.47±1.96 b	4482±125.05 b
**AG 8700 PRO3**	16.86±0.18 a	26.83±0.43 b	195.61±5.25 a	5647±170.88 a

Means followed by distinct lowercase letters, in the columns, show significant differences (LSD test, p ≤ 0.05).

**Fig 2:**
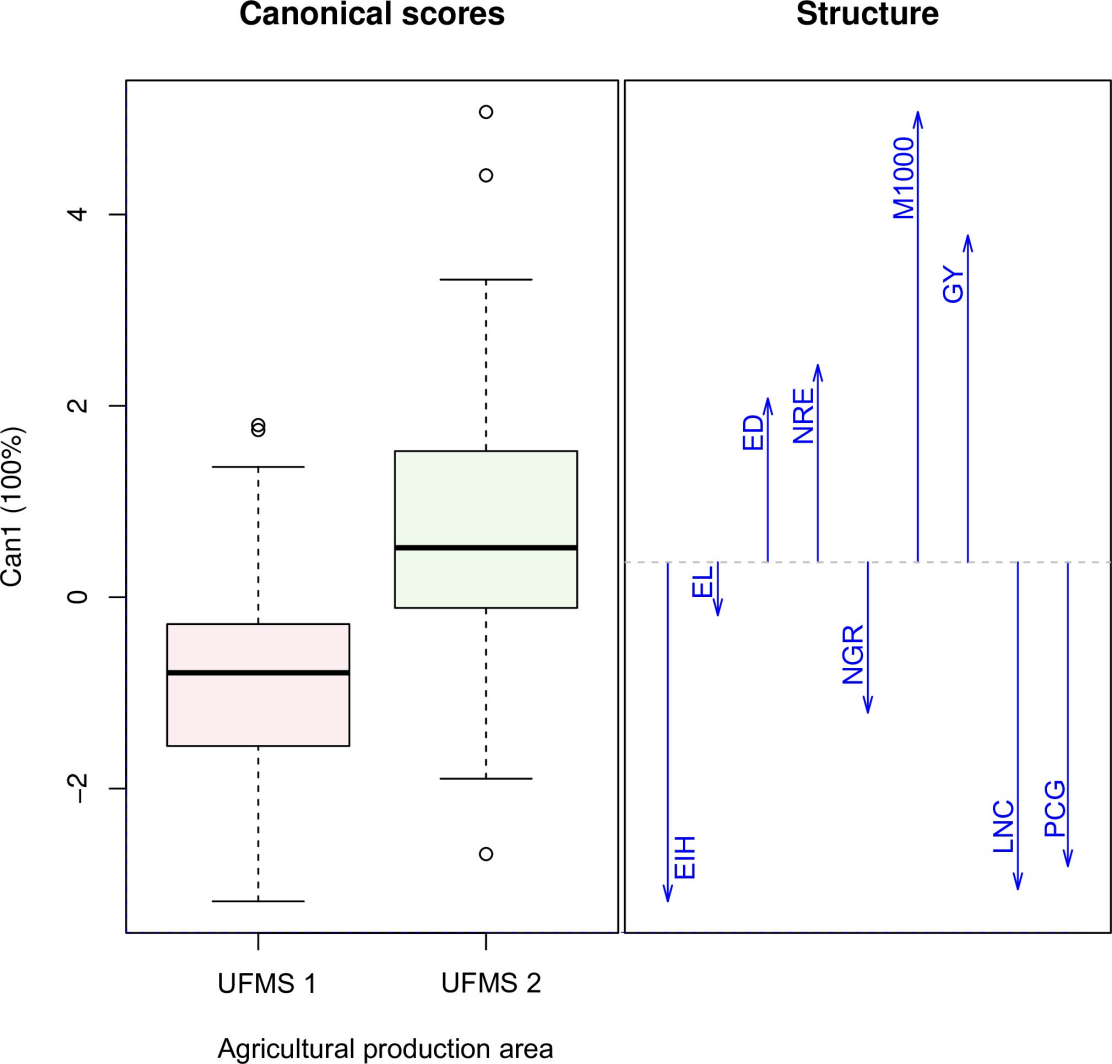
Canonical correlations between the corn agronomic traits and agricultural production areas of medium (UFMS 1) and high (UFMS 2) fertility. Abbreviations: EIH: ear insertion height; EL: ear length; ED: ear diameter; NRE: number of grain rows per ear; NGR: number of grains per row; M1000: mass of one thousand grains; GY: grain yield; LNC: leaf nitrogen concentration; PCG: protein concentration in the grains.

The medium fertility agricultural area (UFMS 1) resulted in an increase in ear insertion height, ear length, number of grains per row, leaf nitrogen concentration, and grain protein concentration, while the high fertility agricultural area (UFMS 2) resulted in an increase in the ear diameter, number of grain rows per ear, 1,000-grain mass and grain (Fig 2). These results confirm those reported by Simão et al. [14], Resende et al. [15], and Baum et al. [34], which showed that the grain yield of the corn crop is directly associated with the level of soil fertility in the agricultural production system. Therefore, the increase in grain yield in the UFMS 2 agricultural area is related to the greater availability of nutrients in this production system, especially P, Ca, Mg and K when compared to the UFMS 1 agricultural area (Table 1). It should be emphasized that, for example, calcium, magnesium, and phosphorus have a direct effect on the photosynthesis process in plants [32]. Therefore, the greater availability of these nutrients in the soil of the UFMS 2 agricultural area resulted in an increase in the photosynthetic rate of corn plants. In turn, this higher production of photoassimilates increased the number of grains per ear (corn rows per ear) and grain filling (thousand-grain mass) and, consequently, the higher grain yield of the crop (Fig 2).

**Fig 3:**
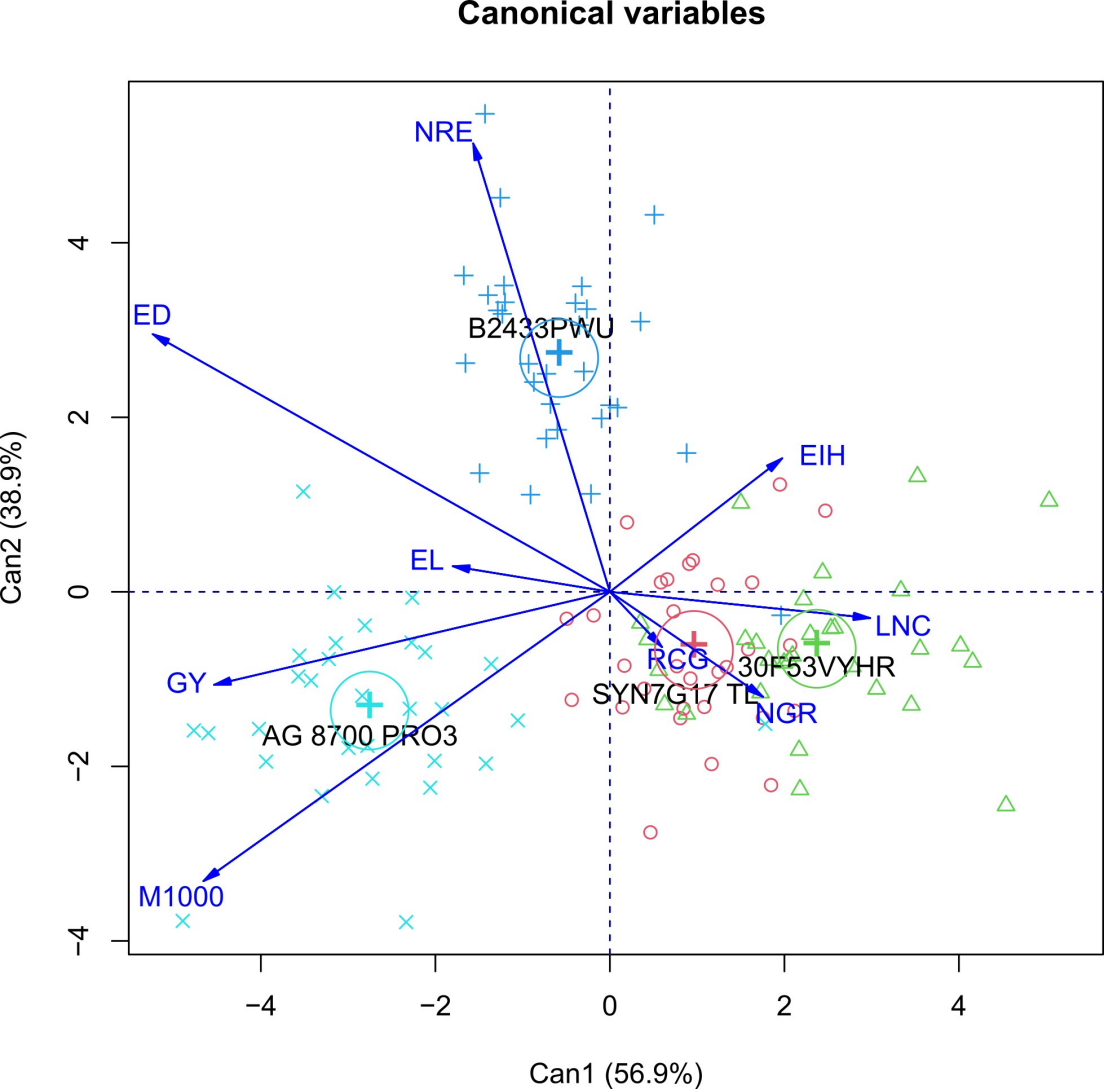
Canonical correlation analysis (CCA) between the crop agronomic traits and four corn cultivars. The blue lines show the canonical correlation between the centroids of the first pair of canonical variates and the linear tendency line. Abbreviations: EIH: ear insertion height; EL: ear length; ED: ear diameter; NRE: number of grain rows per ear; NGR: number of grains per row; M1000: mass of one thousand grains; GY: grain yield; LNC: leaf nitrogen concentration; PCG: protein concentration in the grains.

**Fig 4:**
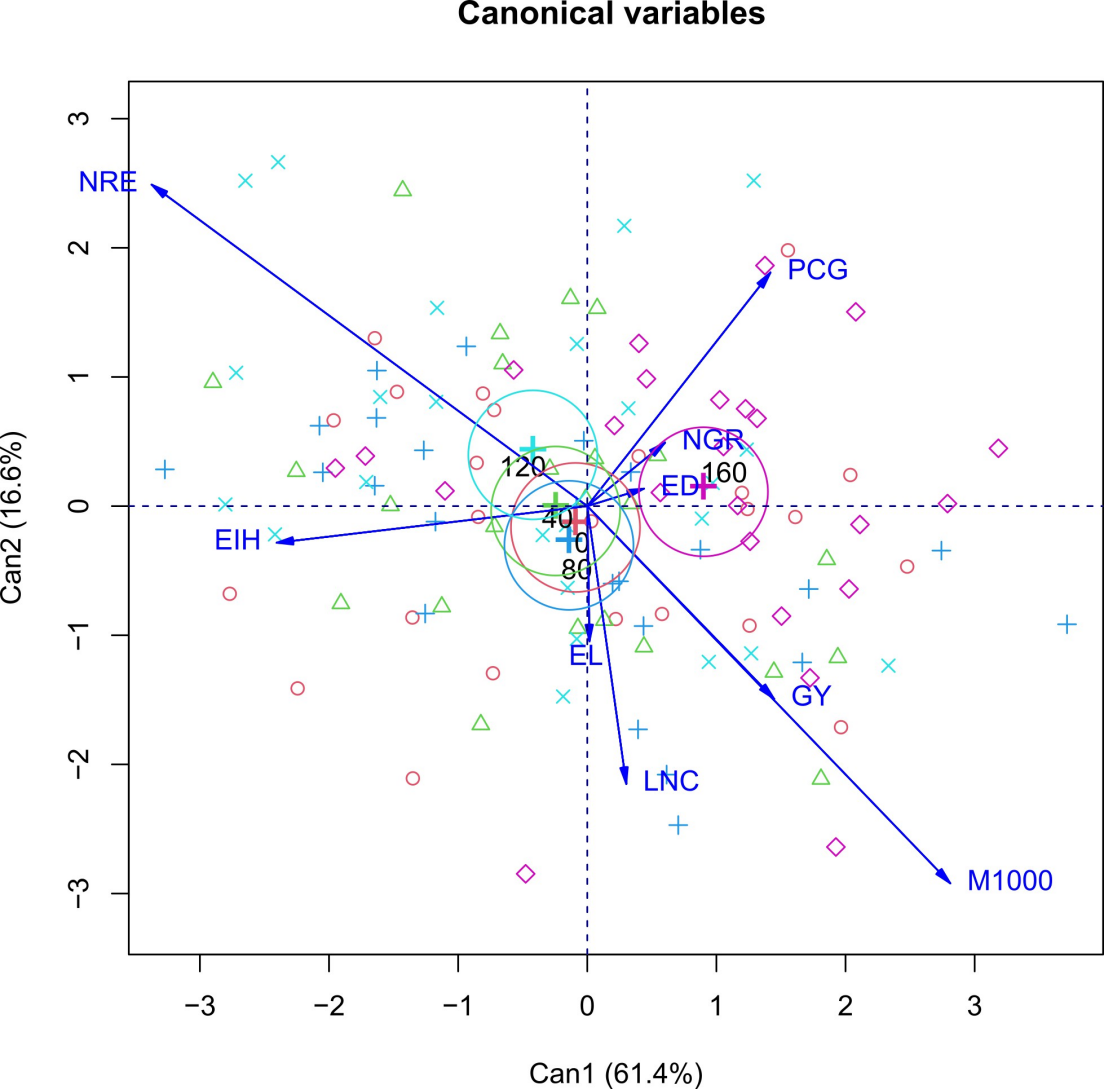
Canonical correlation analysis (CCA) between the corn agronomic traits and N fertilizer application rates. The blue lines show the canonical correlation between the centroids of the first pair of canonical variates and the linear tendency line. Abbreviations: EIH: ear insertion height; EL: ear length; ED: ear diameter; NRE: number of grain rows per ear; NGR: number of grains per row; M1000: mass of one thousand grains; GY: grain yield; LNC: leaf nitrogen concentration; PCG: protein concentration in the grains.

Canonical correlation analysis (CCA) was used to verify the contribution of each dependent variable measured in the off-season corn crop as affected by cultivars (Fig 3) and N fertilization rates (Fig 4). For scores to be represented in a two-dimensional graph, the percentage of retained variance must be higher than 80% [35]. In this study, variances accumulated in the two main canonical variables were 95.8% (Fig 3) and 78.0% (Fig 4), respectively, for each graph, allowing an accurate interpretation.

The cultivar B2433PWU increased the number of grain rows per ear, while the cultivars SYN7G17 TL and 30F53VYHR had higher leaf N concentrations, number of grains per row, and grain protein contents (Fig 3). The cultivar AG 8700 PRO3 had the highest thousand-grain mass and grain yield. All corn cultivars used in this study are simple hybrids and resistant to insects of the order Lepidoptera. However, the cultivar AG 8700 PRO3 had higher grain yield potential than the other cultivars. The grain yield of cultivar AG 8700 PRO3 was 42%, 40%, and 26% higher than the grain yield of the 30F53 VYHR, SYN7G17 TL, and B2433PWU cultivars respectively (Table 9).

The 30F53VYHR and SYN7G17 TL cultivars had higher grain protein concentrations (Fig 4) due to the lowest grain yield of this cultivar (Table 9). Such results are related to the intrinsic characteristics of each genotype and its interaction with the production environment. The phenotype of an individual is influenced by the genotype, which is the individual’s genetic constitution, and by the growing environment, which can be defined as the set of conditions that affect the growth and development of the organism. The genotype and environment interactions can be understood as the different behavior of genotypes when cultivated in contrasting environments. However, these interactions can be simple or complex [31]. The growth and development of corn and, consequently, the grain yield and grain protein content result from the interaction between the cultivars and factors arising from the environment, with the sensitivity to air temperature that induces flowering.

## Conclusions

The crop residues remaining on the soil surface from soybeans not harvested and inoculated with *Bradyrhizobium* spp. can supply most of the nitrogen requirement of off-season corn grown in succession, especially in tropical soils of medium fertility. However, in high fertility tropical soils, the maximum grain yield potential of off-season corn cultivars can be obtained with the application of mineral nitrogen fertilizer in supplement the amount of nitrogen released from unharvested soybean residues. Therefore, the nitrogen requirement depends on the corn cultivar and the agricultural production area. However, our results show that when off-season corn is grown on unharvested soybean residues, nitrogen fertilization in topdressing can be dispensed. The agricultural area with high fertility soil (UFMS 2) enhances the grain yield of the off-season corn crop. The corn cultivar AG 8700 PRO3 has a higher thousand-grain mass and high grain yield potential under Brazilian Cerrado conditions.

## Supporting information

S1 Data (XLSX)
